# Clinical results of autologous fat transfer for basal thumb arthritis with a minimum of three years’ follow-up

**DOI:** 10.1302/2633-1462.512.BJO-2024-0085.R2

**Published:** 2024-12-11

**Authors:** Elisabeth M. Haas-Lützenberger, Iana Emelianova, Moritz C. Bader, Sinan Mert, Nicholas Moellhoff, Wolfram Demmer, Ursula Berger, Riccardo Giunta

**Affiliations:** 1 Department of Hand, Plastic and Aesthetic Surgery, Ludwig-Maximilians-University Munich, Munich, Germany; 2 Institute for Medical Information Processing, Biometry, and Epidemiology, Ludwig-Maximilians-University Munich, Munich, Germany

**Keywords:** Basal thumb osteoarthritis, TMC-OA, Autologous fat, Lipofilling, Regenerative joint therapy, Rhizarthrosis, thumb arthritis, QuickDASH, Wilcoxon tests, strength, patient-reported outcome measures (PROMs), Disabilities of the Arm, Shoulder and Hand questionnaire, transplantation, osteoarthritis (OA), resection arthroplasty, immobilization

## Abstract

**Aims:**

In the treatment of basal thumb osteoarthritis (OA), intra-articular autologous fat transplantation has become of great interest within recent years as a minimally invasive and effective alternative to surgical intervention with regard to pain reduction. This study aims to assess its long-term effectiveness.

**Methods:**

Patients diagnosed with stage one to three OA received a single intra-articular autologous fat transplantation. Fat tissue was harvested from the abdomen and injected into the trapeziometacarpal (TMC) joint under radiological guidance, followed by one week of immobilization. Patients with a minimum three-year post-procedure period were assessed for pain level (numerical rating scale), quality of life (Mental Health Quotient (MHQ)), the abbreviated version of the Disabilities of Arm, Shoulder and Hand questionnaire (QuickDASH)), and grip and pinch strength, as well as their overall impression of the treatment. Wilcoxon tests compared data from pre-intervention, and at one and three years post-intervention.

**Results:**

Out of 136 treated joints, the study involved 87 patients (37 patients were loss to follow-up, and 12 patients (9%) who underwent resection arthroplasty) with a median follow-up of 4.9 years (IQR 5.4 to 5.9). Pain, both at rest and during stress, significantly improved at one year and remained stable through three years. Sex, age, and stage of disease were not associated with postoperative pain levels. Patient-reported outcome measures for QuickDASH and MHQ improved up to at least three years post-treatment. Patients reported high satisfaction and willingness to recommend the procedure. Grip and pinch strength did not significantly change over time.

**Conclusion:**

The data show that autologous fat transfer has a longer-lasting effect in two-thirds of re-examined patients. If patients had an initial positive response, the pain-reducing effect lasted for at least three years. Therefore, this minimally invasive approach can offer a valuable treatment alternative for basal thumb OA.

## Introduction

Trapeziometacarpal osteoarthritis (TMC-OA) is one of the most common degenerative diseases of the hand; elderly females are the most affected. Definitive surgical treatments require a long rehabilitation period, so patients often opt for minimally invasive techniques. Although total replacement of the TMC joint has shown impressive results in terms of faster recovery, less pain, and better thumb opposition, the high risk of complications should not be underestimated, and trapeziectomy remains the “gold” standard in the surgical treatment of TMC-OA.^1^

Nevertheless, deferring surgery is of great interest, particularly from an economic- and patient-centered perspective. Intra-articular injection of autologous fat has increasingly emerged as a minimally invasive and effective treatment option for TMC-OA. The required adipose tissue is easily accessible and available in large quantities. Long-term follow-up has shown that there is no risk of malignant potential with autologous fat grafting. Autologous fat transfer (AFT) is increasingly being used in the treatment of OA because of its good clinical results.^2-4^ This has also been shown to improve joint cartilage in animal studies.^5^ In humans, autologous fat is applied both to the lower limb, mainly to the knee and ankle joint, and to the upper limb, mainly to the TMC and radiocarpal joint. The primary clinical outcome is a significant reduction in pain, resulting in an extremely high level of patient satisfaction. In addition, AFT aims to postpone definitive surgery, slow the progression of OA, and achieve a better quality of life (QoL).

Many studies have demonstrated the positive and lasting effects of the “single shot” therapy, but there is still a great need for long-term results. Therefore, we re-examined the patients from our previous one-year study group and included those whom we were able to contact after that period, who had more than three years of follow-up in this study.^2^

## Methods

In our preliminary investigation, patients with stage one to three basal thumb OA according to the Eaton-Littler classification^6^ were included following unsuccessful conservative therapy for at least six months, such as physiotherapy, splinting, non-steroidal anti-inflammatory drugs, or corticosteroid injection, with the last injection being at least three months prior to ensure its effects were ruled out. We recommended AFT to all patients as the first minimally invasive approach before a conversion to definitive surgery. All patients preferred AFT as a first attempt. Patients with a history of thumb surgery were not included. We conducted an intra-articular autologous fat transplantation to the TMC joint without any alterations or additional substances. Autologous fat was harvested from the lower abdomen and injected immediately after mechanical homogenization in the TMC joint under fluoroscopic radiological guidance. The patients were placed in a splint for a week, and no additional therapy was administered. The procedure was described in detail in our initial publication.^2^

To examine the extended follow-up period, we included all patients with a minimum of three years’ post-intervention and invited them for a follow-up examination.

In all, 136 joints (128 patients) were initially included in the first study, which fulfilled the inclusion criteria. Overall, 37 patients (29%) were lost to follow-up, and 12 patients (9%) underwent resection arthroplasty, from whom we did not have any further follow-up data. Thus, a total of 87 joints were included in the long-term follow-up examination, and these were subsequently categorized into responder and non-responder groups. The main outcome measure for pain was assessed using the numerical rating scale (NRS) during rest and stress, since pain is the most important criterion for QoL.

Secondary outcome parameters were the QoL determined by the patient-reported outcome measures (PROMs): the Michigan Hand Outcomes Questionnaire (MHQ);^7^ and the abbreviated version of the Disabilities of the Arm, Shoulder and Hand questionnaire (QuickDASH).^8^ The definition of “responder” was a reduction of pain of minimum three points on the NRS. Alongside patients’ grip strength (Jamar Dynamometer; Jamar Technologies, USA) and pinch strength (Pinch Gauge Dynamometer PG-30; B&L Engineering, USA), there were also responses to two questions: whether the patients rated the procedure as a success (“definitely”, “slightly”, or “none”); and whether they would recommend the procedure. The analysis compared the change in outcomes after one year and at least three years to explore long-term effectiveness.

### Demographic analysis/patients

Overall, 61 female cases (70.1%) and 26 male cases (29.8%) with a mean age of 61.2 years (SD 9.7; 29 to 79) and with a mean BMI of 24.4 kg/m^2^ (SD 4.7; 18.4 to 44.2) were included in this study. A total of 79 patients and 87 cases attended the three-year follow-up visit. The median follow-up period was 4.9 years (IQR 4.1 to 5.3) (Table I).

**Table I.** Demographic data of patients.

Variable	Data
**Sex, n (%)**	
Male	26 (29.9)
Female	61 (70.1)
Mean age, yrs (range)	61.2 (29 to 79)
Mean BMI, kg/m/^2^ (SD; range)	24.4 (4.7; 18.4 to 44.2)
**Operated hand, n (%)**	
Dominant	39 (45)
Non-dominant	48 (55)
**Dominant hand, n (%)**	
Right	80 (92)
Left	7 (8)
**Eaton & Littler stage, n (%)**	
1	54 (62)
2	26 (30)
3	7 (8)
Median follow-up, yrs (IQR)	4.9 (4.1 to 5.3)

Most patients (92%) were right-handed. In 45% of the cases, the dominant hand was affected. According to the Eaton and Littler classification, most affected hands (62%, n = 54) showed advanced OA (stage 3), 30% (n = 26) had stage two OA, and only 8% (n = 7) had mild OA (stage 1).^9^

### Statistical analysis

All data were documented in a REDCap data base (Research Electronic Data Capture; Vanderbilt University, USA), all analyses were performed using the statistical computing software R v. 3.6.2 (R Foundation for Statistical Computing, Austria). Distribution of outcomes are presented in boxplots, and changes between follow-up are tested employing Wilcoxon tests signed-rank test for matched samples. Univariate logistic regression models were used to explore the impact of age, sex, BMI, stage, and preoperative NRS stress on the chances of treatment success (i.e. showing a reduction in NRS stress of at least three score points).

## Results

### Primary outcome parameter: pain

**Fig. 1:**
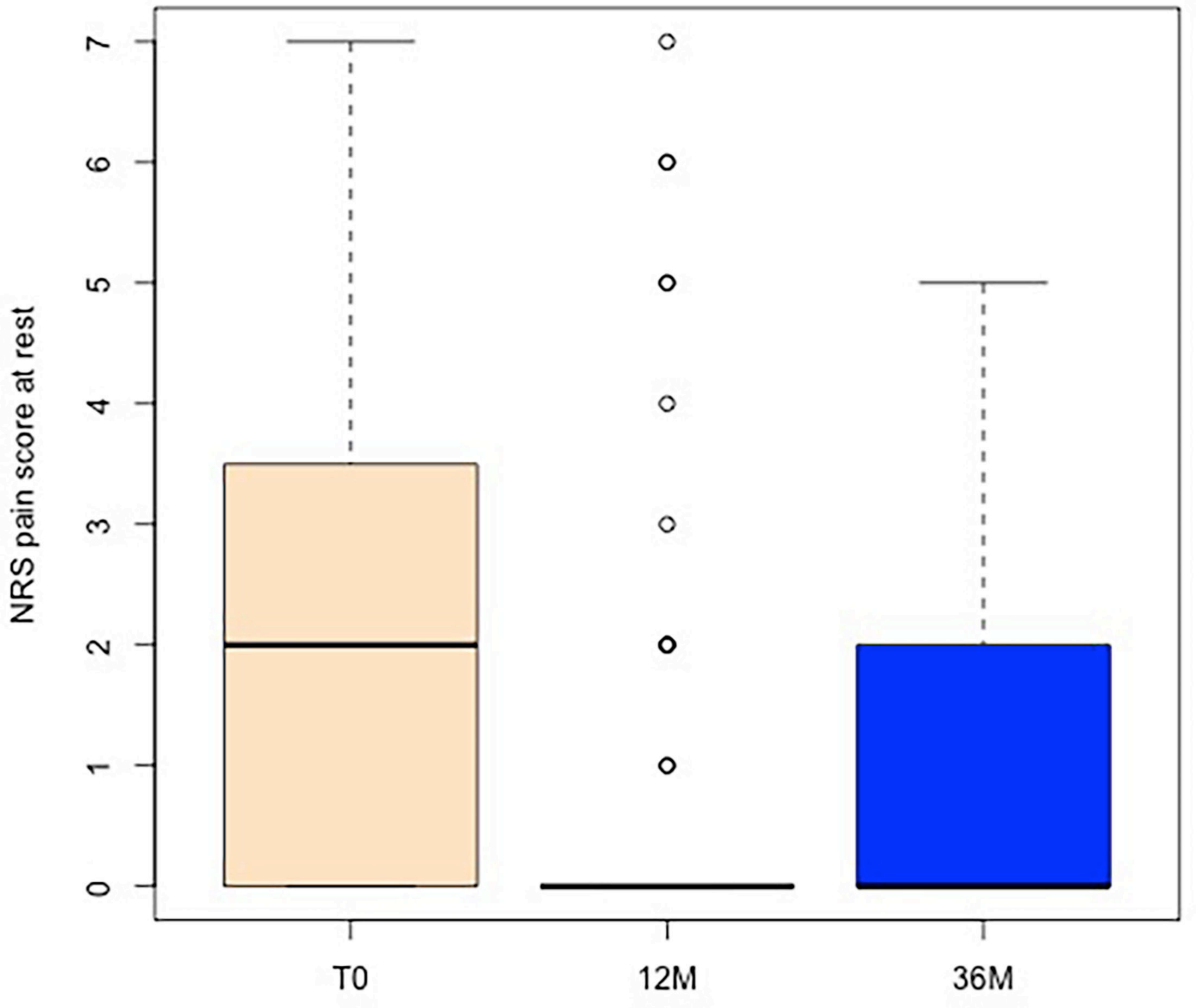
Numerical rating scale (NRS) of the operated hand while resting, with boxplot showing the absolute preoperative values: T0, and at follow-up after 12 months and 36 months.

**Fig. 2:**
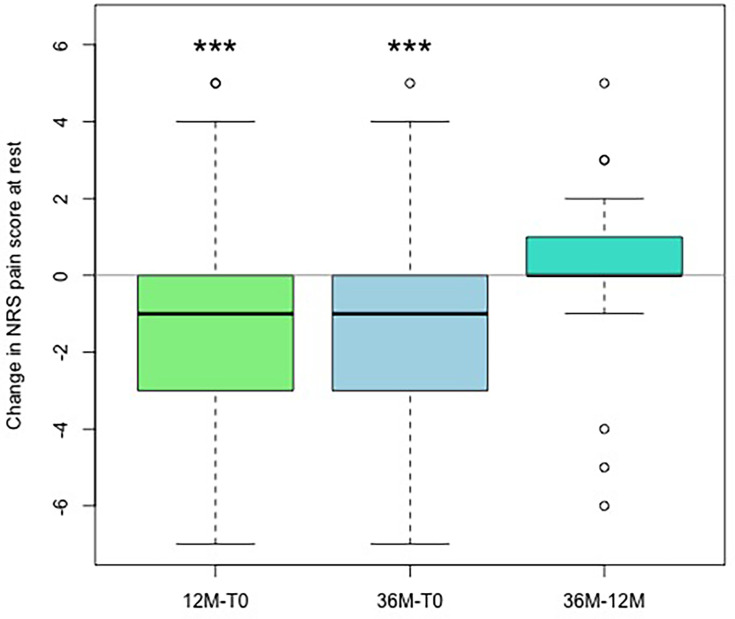
Changes in numerical rating scale (NRS) of the operated hand while resting, with boxplot showing the differences comparing two timepoints: baseline (T0), with follow-up at 12 months and at 36 months, and changes between 12 and 36 months. One- and three-year follow-up show significant correlation when compared to baseline. *p < 0.05, **p < 0.005, ***p < 0.001; Wilcoxon test.

Figure 1 displays the NRS distribution of the operated hand during rest at baseline (T0), and after months 12 and 36. Figure 2 shows the changes in NRS in comparison to baseline and between the two follow-up time periods. A significant improvement of NRS at rest after year one and after long-term follow-up over three years when compared to baseline (p = 0.002 and p = 0.003, Wilcoxon test) was found. In addition, there was no significant worsening of NRS between year one and year three (p = 0.285). Overall, 33% of cases were responders to treatment and showed a reduction of three points on the NRS at 36 months compared to T0, while another 33% showed a minimal improvement of one or two points in pain. At the last follow-up visit of three years, 94% of cases had no or only mild pain (maximum three points on NRS).

**Fig. 3:**
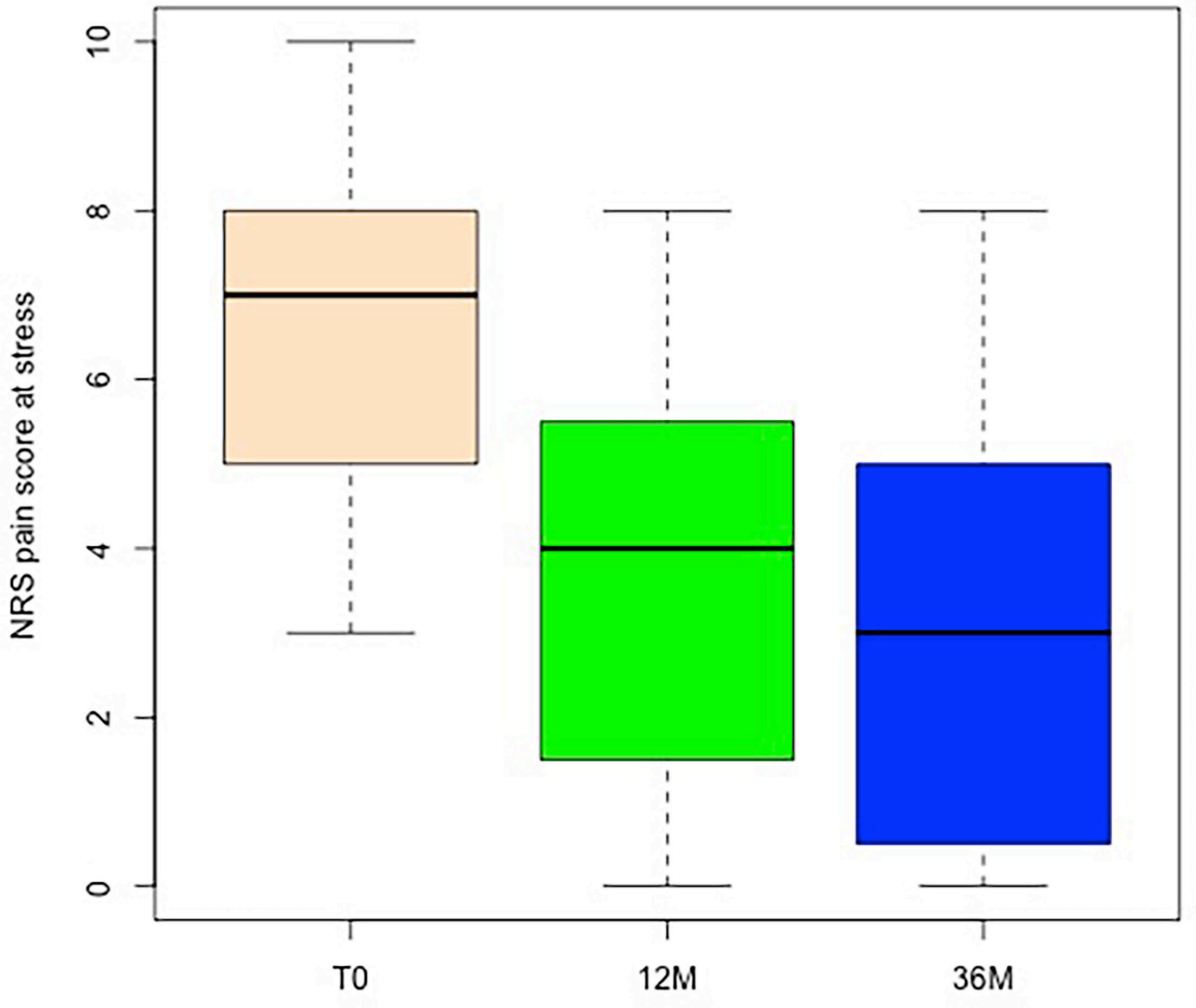
Numerical rating scale (NRS) of the operated hand at stress, with boxplot showing the absolute values preoperatively: T0 (beige), and at follow-up after 12 months and 36 months.

**Fig. 4:**
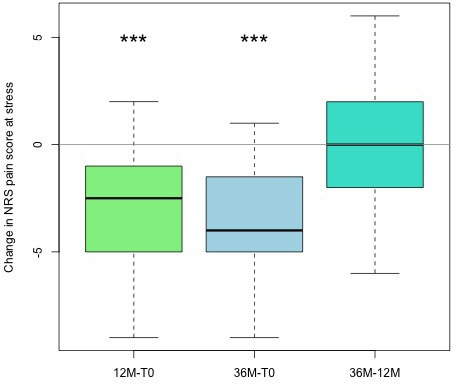
Changes in numerical rating scale (NRS) of the operated hand at stress, with boxplot showing the differences comparing two timepoints: baseline (T0), with follow-up at 12 months and at 36 months, and changes between months 12 and 36. *p < 0.05, **p < 0.005, ***p < 0.001; Wilcoxon test.

Pain during stress improved significantly when comparing baseline to one- and three-year follow-up (p < 0.001, Wilcoxon test). No difference was observed between the one- and three-year follow-up periods (p = 0.855; Wilcoxon test, Figure 3 and Figure 4). Under stress, 52% cases indicated only mild pain (NRS scale 3 or lower). Thus, a total of 66% of cases demonstrated a reduction of three points on the NRS at 36 months compared to T0, and can thus be considered as responders to treatment. Chance of respondence was only associated with pain scores (NRS) at stress at T0 (preoperative), where higher scores increased chances of a treatment respondence. Sex, age at intervention, and stage of disease did not show any significant association with chances of improvement (Table II). BMI showed a negative trend decreasing chances of success by 18% per increase of one unit in BMI (p = 0.052, univariate logistic regression and Wald test, Table II).

**Table II.** Results of logit regression.

Characteristics	OR	p-value*
Eaton & Littler stage	0.93	0.874
BMI, kg/m^2^	0.82	0.052
Age at OP, yrs	0.99	0.858
Male sex	4.79	0.061

* Univariate logistic regression with Wald test.

OR, odds ratio.

### Secondary outcome parameters

**Fig. 5:**
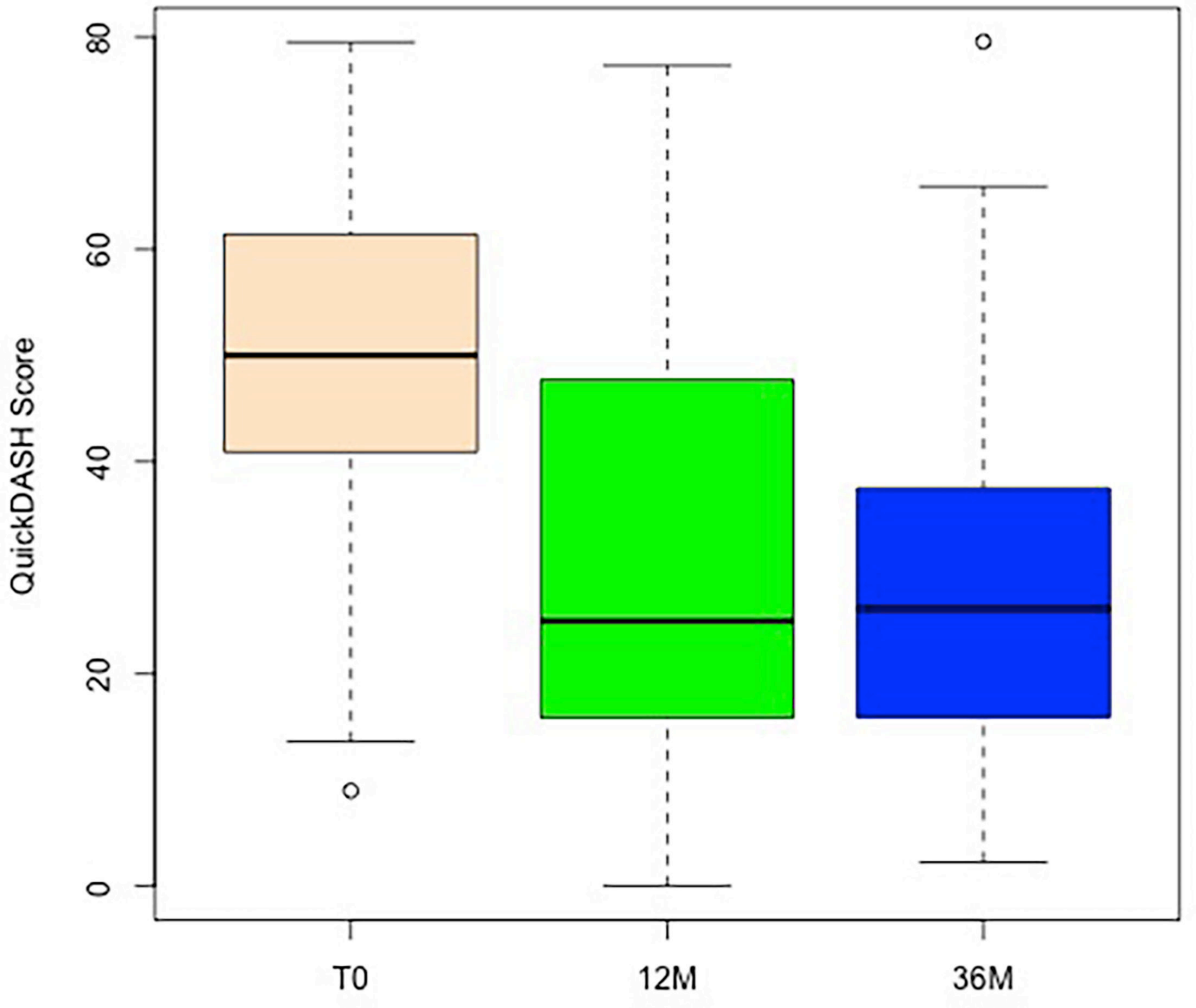
Boxplot showing the absolute abbreviated version of the Disabilities of the Arm, Shoulder and Hand questionnaire (QuickDASH) values preoperatively: T0, and at follow-up after 12 months and 36 months.

**Fig. 6:**
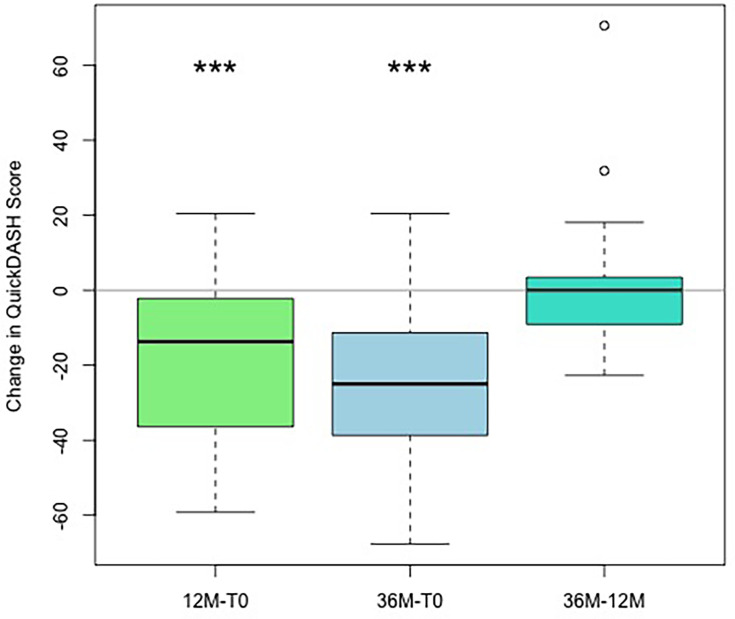
Changes in abbreviated version of the Disabilities of the Arm, Shoulder and Hand questionnaire (QuickDASH), with boxplot showing the differences comparing two timepoints: baseline (T0), with follow-up at 12 months and at 36 months, and changes between months 12 and 36. *p < 0.05, **p < 0.005, ***p < 0.001, Wilcoxon test.

Figure 5 illustrates the QuickDASH absolute scores at three timepoints under stress. The initial follow-up score at T12 demonstrated a significant decrease when compared to T0 value (p < 0.001, Wilcoxon test). This reduction persisted throughout the three-year follow-up period, as demonstrated by the follow-up score after three years in comparison to the preoperative value (p < 0.001, Wilcoxon test), with no significant alteration during the follow-up period (one to three years) (p = 0.773, Wilcoxon test; Figure 6).

**Fig. 7:**
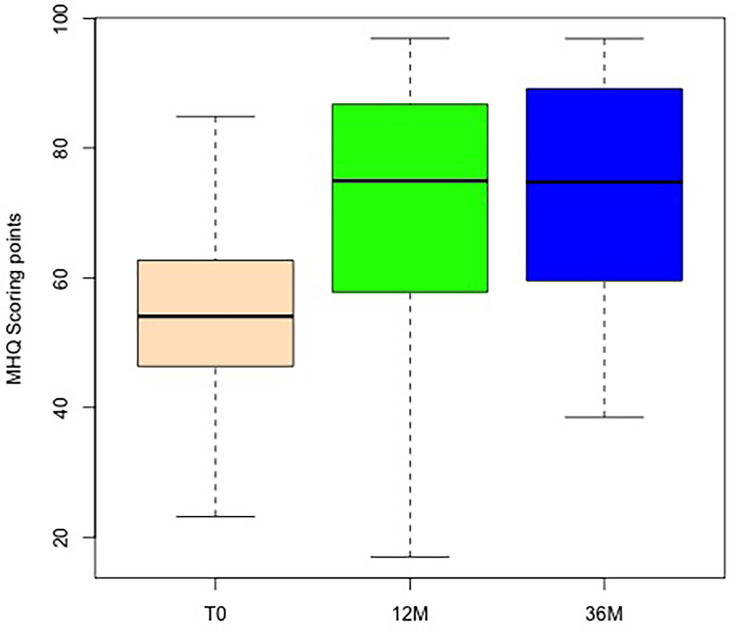
Results of the Mental Health Quotient (MHQ) with respect to the operated hand, with boxplot showing the absolute values preoperatively: T0 (beige), and at follow-up after 12 months and 36 months.

**Fig. 8:**
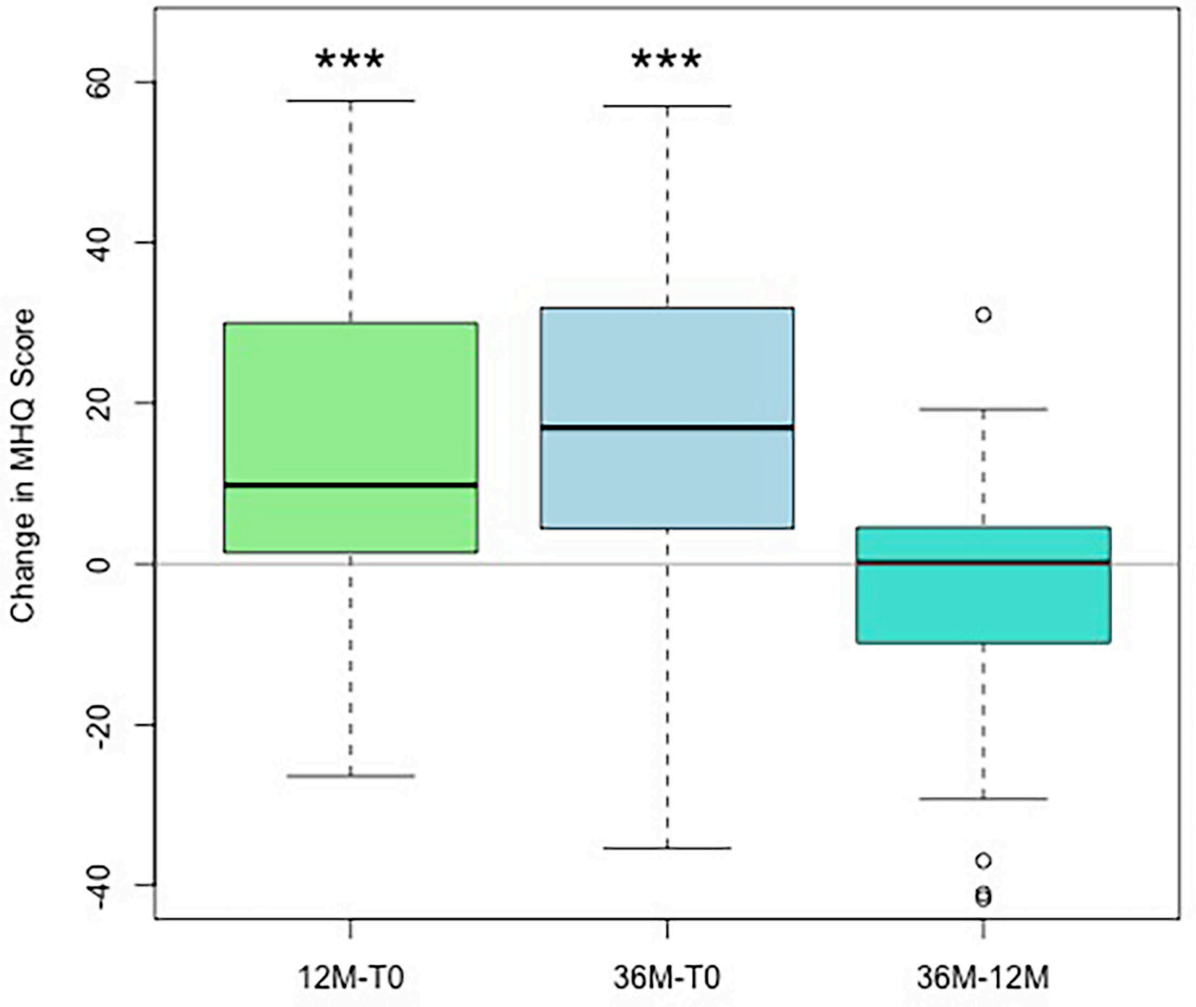
Changes in Mental Health Quotient (MHQ) with regard to the operated hand, with boxplot showing the differences comparing two timepoints: baseline (T0), with follow-up at 12 months and at 36 months, and changes between months 12 and 36. *p < 0.05, **p < 0.005, ***p < 0.001; Wilcoxon test.

The analysis of the MHQ demonstrated a substantial increase during the first follow-up after one year and at the second follow-up after three years (Figure 7). Therefore, a significant difference was observed between one and three years (p < 0.001, Wilcoxon test). The median score remained the same (zero change) when compared with follow-up T1 (one year) and T12 (three years) (Figure 8).

In terms of patients’ subjective evaluation, 53 patients (61%) noticed a marked improvement, 15 (13%) observed a slight improvement, and 30 (26%) did not observe any changes after one year. The answers only changed slightly after three years, with 58 patients (66%) noticing a definite therapy success, 20 (23%) some positive effect, and ten (11%) not seeing any improvement.

Nevertheless, 64 patients (73%) after one year and 71 (82%) after three years would undergo the surgery again. Furthermore, 71 patients (84%) reported that they would recommend the procedure after one year, and 79 (91%) stated they would recommend it after three years.

The mean pinch strength at T0 was 5.9 kg, and remained unchanged (5.9 kg after 12 months and 5.8 kg after 36 months; Supplementary Figure a). The mean grip strength was 28.4 kg preoperatively (T0), 27.9 kg after one year, and 23.7 kg after three years. Statistical analysis revealed no significant changes throughout the different timepoints (Supplementary Figure b).

## Discussion

Basal thumb arthritis has a high incidence in the elderly population, particularly among females.^10^ Many conservative and operative treatments have been described. The role of regenerative therapies and minimally invasive techniques has become more popular over time. AFT has played a tremendous role in the field of plastic and hand surgery. A number of clinical applications for a regenerative approach have been reported in recent years.^11^ One-third of studies on the use of autologous fat cells pertains to treating musculoskeletal disorders. Numerous publications describe the positive impact of injecting abdominal fat sourced from the same patient into a joint, including randomized controlled trials (RCTs) with extended follow-up periods.^12-15^ Most clinical studies have focused on treating knee OA, as it is the most prevalent degenerative OA of the human body.^16^

A recently published animal study applying adiposed-derived stem cells to the knees of rats has revealed a substantial decrease in cartilage damage related to OA.^17^ Freitag et al^13^ demonstrated a noteworthy decrease in pain and an enhanced ability to carry out daily activities. AFT has been applied to the TMC joint for over ten years, with convincing short-term results reported in the literature.^4,18-20^ Our cohort provides the most extensive data on patients suffering from basal thumb OA. Although Herold et al^20^ confirmed our results after five years, they followed up with only 42 patients. Their data showed a significant reduction in pain on NRS from 5.9 to 1.7; an equivalent reduction was observed for the DASH score. Froschauer et al^18^ reported analogous findings with a median follow-up of 31 patients over five years and a pain decrease of six points (from 7 to 1) on the visual analogue scale. They also presented a sustained pain reduction and QoL improvement, which align with our results. Our longer-term outcomes indicate promising potential for sustained effectiveness when patients responded in the initial months post-treatment. In our previously published data from the one-year follow-up, we were unable to examine the therapy’s impact on strength. However, measurements taken after three years confirm that the therapy does not have a significant impact on strength.^2^

Our study has several limitations. First, we excluded patients who underwent surgery within the first year after treatment due to missing data. Second, we have an additional percentage of patients lost to follow-up. This issue arose partly because our study began in 2014, and contact information for some patients was outdated, making them unreachable. In addition, some patients were from distant locations, and we were unable to offer compensation for their travel. Furthermore, the follow-up period coincided with the COVID-19 pandemic, which likely contributed to the attrition. A third limitation pertains to our study’s design. While RCTs provide the highest level of scientific evidence, our retrospective study lacked a control group, which is a significant weakness. However, in a previous study, we included a corticosteroid control group that showed no effect after just six weeks, leading to the discontinuation of that study design.^21^ Despite these limitations, our findings underscore the necessity of conducting future studies with robust, controlled designs.

Until now, the exact cause of the positive effect on pain reduction has not been fully understood. While some ideas suggest a mechanical reduction of bone-to-bone contact, thereby reducing direct abrasive contact of the joint surfaces, others point towards an immunomodulatory and chondroprotective effect through paracrine secretion, resulting in an anti-inflammatory effect. Potentially, there may be a regenerative component that can decelerate the progression of OA. The anti-inflammatory effect can be attributed to the reduction of pro-inflammatory cytokines and growth factors, such as matrix metalloproteinase, tumour necrosis factor-alpha, and interleukin-1, which cause the development of OA.^21^ In an experimental study, Taha et al^22^ were able to provide support for this thesis through an analysis of gene sequencing. Nevertheless, basic science studies are needed to further explain the specific pathomechanism.

The crucial aspect is patient satisfaction, which results from pain reduction. Our findings validate patient satisfaction through a marked decrease in QuickDASH score and increased MHQ.

AFT is merely one of numerous minimal invasive intra-articular therapies available. Its superiority over other applications has yet to be substantiated by evidence, although a few clinical trials compare competing techniques. The enduring impact of AFT, when compared to the standard of care (corticosteroid injection), is documented in a series of case reports.^23^ Another intra-articular substance that can be used for OA treatment is hyaluronic acid (HA). Being a natural component of the joint, the synthetic provision acts as a substitute for the degradation caused by OA. Nevertheless, its effectiveness has not yet been convincingly established through clinical trials, and thus, its significance in OA treatment is debatable.^24^ Platelet-rich plasma (PRP) is a widely discussed agent. It is used not only in the therapy for OA, but also in various other regenerative treatments.^25^ The positive impact of PRP can be explained by its containment of growth factors and anti-inflammatory cytokines.^26^ All three methods (AFT, HA, and PRP) lack high-quality evidence supporting their efficacy. Only one systematic review and meta-analysis examines the differences between PRP and AFT: according to Winter et al,^27^ both therapies demonstrate a lasting effect in reducing pain, and both are superior to corticosteroids.

The beneficial impact of AFT has been recognized and used for many years. Presently, ongoing clinical trials are examining the efficacy of platelet-rich-plasma in the treatment of OA. Therefore, in consideration of safety factors, traditional fat grafting is advised.^27^

Nevertheless, the clinical outcome and patient satisfaction, with an improvement in QoL of over 50%, justify including AFT in the therapeutic algorithm for the treatment of basal thumb arthritis. This is especially supported by our results, which demonstrate that the positive effect lasts for at least three years. The rapid onset of pain reduction is a significant advantage compared to conventional surgical treatment options such as arthroplasties.^28^ More research is needed to evaluate the long-term effectiveness of the therapy, as are RCTs in order to provide robust evidence.

In conclusion, the incidence of basal thumb arthritis is on the rise, particularly among the elderly female population. Consequently, there is a strong demand for a less invasive therapy compared to surgery, but with longer-lasting effects than standard injection therapies. Our comprehensive results, derived from a large single-arm patient cohort with a follow-up period of at least three years, demonstrate the efficacy of a single-shot AFT. In responders, who make up 66% of our follow-up cohort, the positive effects last for at least three years. One-third of patients reported a significant improvement of pain at rest of at least three points, while one-third reported only a slight improvement in pain at rest. However, the high rate of loss to follow-up (29%) and the number of patients with treatment failure and conversion to surgery (9%) from our initial cohort must be considered.

As if now, no patient-specific characteristics can be defined to predict a positive response. For responders, however, it can be a quick and effective solution to achieve suitable pain levels during daily activities for several years and therefore delay more invasive surgeries. Large-scale randomized placebo-controlled trials are needed to further investigate and assess the efficacy of AFT in the treatment of basal thumb OA.

**Take home message**

- Intra-articular autologous fat transplantation is a minimally-invasive, long-lasting treatment option for basal thumb osteoarthritis.

- The procedure provides significant and sustained pain relief, improves patient-reported outcomes, and is associated with high satisfaction over a follow-up period of at least three years.

- This approach offers a promising alternative to surgical intervention for suitable patients.

## Data Availability

The data that support the findings for this study are available to other researchers from the corresponding author upon reasonable request.

## References

[1] Qureshi MK Halim UA Khaled AS Roche SJ Arshad MS Trapeziectomy with ligament reconstruction and tendon interposition versus trapeziometacarpal joint replacement for thumb carpometacarpal osteoarthritis: a systematic review and meta-analysis J Wrist Surg 2022 11 3 272 278 10.1055/s-0041-1731818 35845236 PMC9276058

[2] Haas EM Eisele A Arnoldi A et al. One-year outcomes of intraarticular fat transplantation for thumb carpometacarpal joint osteoarthritis: case review of 99 joints Plast Reconstr Surg 2020 145 1 151 159 10.1097/PRS.0000000000006378 31592943

[3] Herold C Rennekampff H-O Groddeck R Allert S Autologous fat transfer for thumb carpometacarpal joint osteoarthritis: a prospective study Plast Reconstr Surg 2017 140 2 327 335 10.1097/PRS.0000000000003510 28369017

[4] Kemper R Wirth J Baur E-M Arthroscopic synovectomy combined with autologous fat grafting in early stages of CMC osteoarthritis of the thumb J Wrist Surg 2018 7 2 165 171 10.1055/s-0037-1604045 29576924 PMC5864490

[5] Yang W-T Ke C-Y Yeh K-T et al. Stromal-vascular fraction and adipose-derived stem cell therapies improve cartilage regeneration in osteoarthritis-induced rats Sci Rep 2022 12 1 2828 10.1038/s41598-022-06892-3 35181731 PMC8857326

[6] Eaton EG Glickel SZ Trapeziometacarpal osteoarthritis: staging as a rationale for treatment Hand Clin 1987 3 455 471 10.1016/S0749-0712(21)00761-7 3693416

[7] Chung KC Pillsbury MS Walters MR Hayward RA Reliability and validity testing of the michigan hand outcomes questionnaire J Hand Surg Am 1998 23 4 575 587 10.1016/S0363-5023(98)80042-7 9708370

[8] Beaton DE Wright JG Katz JN Upper Extremity Collaborative Group Development of the QuickDASH: comparison of three item-reduction approaches J Bone Joint Surg Am 2005 87-A 5 1038 1046 10.2106/JBJS.D.02060 15866967

[9] Eaton RG Glickel SZ Trapeziometacarpal osteoarthritis. Staging as a rationale for treatment Hand Clin 1987 3 4 455 471 3693416

[10] Neogi T Zhang Y Epidemiology of osteoarthritis Rheum Dis Clin North Am 2013 39 1 1 19 10.1016/j.rdc.2012.10.004 23312408 PMC3545412

[11] Lee S Chae D-S Song B-W et al. ADSC-based cell therapies for musculoskeletal disorders: a review of recent clinical trials Int J Mol Sci 2021 22 19 10586 10.3390/ijms221910586 34638927 PMC8508846

[12] Song Y Du H Dai C et al. Human adipose-derived mesenchymal stem cells for osteoarthritis: a pilot study with long-term follow-up and repeated injections Regen Med 2018 13 3 295 307 10.2217/rme-2017-0152 29417902

[13] Freitag J Bates D Wickham J et al. Adipose-derived mesenchymal stem cell therapy in the treatment of knee osteoarthritis: a randomized controlled trial Regen Med 2019 14 3 213 230 10.2217/rme-2018-0161 30762487

[14] Lee W-S Kim HJ Kim K-I Kim GB Jin W Intra-articular injection of autologous adipose tissue-derived mesenchymal stem cells for the treatment of knee osteoarthritis: a phase IIb, randomized, placebo-controlled clinical trial Stem Cells Transl Med 2019 8 6 504 511 10.1002/sctm.18-0122 30835956 PMC6525553

[15] Lu L Dai C Zhang Z et al. Treatment of knee osteoarthritis with intra-articular injection of autologous adipose-derived mesenchymal progenitor cells: a prospective, randomized, double-blind, active-controlled, phase IIb clinical trial Stem Cell Res Ther 2019 10 1 143 10.1186/s13287-019-1248-3 31113476 PMC6528322

[16] Cui A Li H Wang D Zhong J Chen Y Lu H Global, regional prevalence, incidence and risk factors of knee osteoarthritis in population-based studies E Clin Med 2020 29–30 100587 10.1016/j.eclinm.2020.100587 34505846 PMC7704420

[17] Yan B Lv S Tong P et al. Intra-articular injection of adipose-derived stem cells ameliorates pain and cartilage anabolism/catabolism in osteoarthritis: preclinical and clinical evidences Front Pharmacol 2022 13 854025 10.3389/fphar.2022.854025 35387326 PMC8978713

[18] Froschauer SM Holzbauer M Wenny R et al. Autologous fat transplantation for thumb carpometacarpal joint osteoarthritis (liparthroplasty): a case series with two years of follow-up J Clin Med 2020 10 1 113 10.3390/jcm10010113 33396314 PMC7795524

[19] Holzbauer M Schmidt M Mihalic JA Duscher D Froschauer SM Liparthroplasty for thumb carpometacarpal joint osteoarthritis: a case series with median 5 years of follow-up J Clin Med 2022 11 21 6411 10.3390/jcm11216411 36362639 PMC9656523

[20] Herold C Lange J Rennekampff HO Allert S Meyer Marcotty M Autologous fat transfer for thumb carpometacarpal joint osteoarthritis: long term results Z Orthop Unfall 2023 161 5 511 515 10.1055/a-1737-4541 35272382

[21] van den Berg WB The role of cytokines and growth factors in cartilage destruction in osteoarthritis and rheumatoid arthritis Z Rheumatol 1999 58 3 136 141 10.1007/s003930050163 10441840

[22] Taha S Akova E Saller MM Giunta RE Haas-Lützenberger EM Early transcriptional changes of adipose-derived stem cells (ADSCs) in cell culture Medicina (Kaunas) 2022 58 9 1249 10.3390/medicina58091249 36143926 PMC9501538

[23] Haas EM Volkmer E Giunta RE Pilot study on the effects and benefits of autologous fat grafting in osteoarthritis of the CMC-1 joint compared to intraarticular cortisone injection: results after 3 months Handchir Mikrochir Plast Chir 2017 49 5 288 296 10.1055/s-0043-120114 29041020

[24] Campbell KA Erickson BJ Saltzman BM et al. Is local viscosupplementation injection clinically superior to other therapies in the treatment ofosteoarthritis of the knee: a systematic review of overlapping meta-analyses Arthroscopy 2015 31 10 2036 2045 10.1016/j.arthro.2015.03.030 25998016

[25] Everts P Onishi K Jayaram P Lana JF Mautner K Platelet-rich plasma: new performance understandings and therapeutic considerations in 2020 Int J Mol Sci 2020 21 20 7794 10.3390/ijms21207794 33096812 PMC7589810

[26] Bendinelli P Matteucci E Dogliotti G et al. Molecular basis of anti-inflammatory action of platelet-rich plasma on human chondrocytes: mechanisms of NF-κB inhibition via HGF J Cell Physiol 2010 225 3 757 766 10.1002/jcp.22274 20568106

[27] Winter R Hasiba-Pappas SK Tuca A-C et al. Autologous fat and platelet-rich plasma injections in trapeziometacarpal osteoarthritis: a systematic review and meta-analysis Plast Reconstr Surg 2023 151 1 119 131 10.1097/PRS.0000000000009789 36219860

[28] Erne HC Cerny MK Ehrl D et al. Autologous fat injection versus lundborg resection arthroplasty for the treatment of trapeziometacarpal joint osteoarthritis Plast Reconstr Surg 2018 141 1 119 124 10.1097/PRS.0000000000003913 28922320

